# A Semantic Transformation Methodology for the Secondary Use of Observational Healthcare Data in Postmarketing Safety Studies

**DOI:** 10.3389/fphar.2018.00435

**Published:** 2018-04-30

**Authors:** Anil Pacaci, Suat Gonul, A. Anil Sinaci, Mustafa Yuksel, Gokce B. Laleci Erturkmen

**Affiliations:** ^1^Software Research & Development and Consultancy Corp., Ankara, Turkey; ^2^David R. Cheriton School of Computer Science, University of Waterloo, Waterloo, ON, Canada; ^3^Department of Computer Engineering, Middle East Technical University, Ankara, Turkey

**Keywords:** semantic transformation, healthcare datasets, common data model, postmarketing safety study, pharmacovigilance

## Abstract

**Background:** Utilization of the available observational healthcare datasets is key to complement and strengthen the postmarketing safety studies. Use of common data models (CDM) is the predominant approach in order to enable large scale systematic analyses on disparate data models and vocabularies. Current CDM transformation practices depend on proprietarily developed Extract—Transform—Load (ETL) procedures, which require knowledge both on the semantics and technical characteristics of the source datasets and target CDM.

**Purpose:** In this study, our aim is to develop a modular but coordinated transformation approach in order to separate semantic and technical steps of transformation processes, which do not have a strict separation in traditional ETL approaches. Such an approach would discretize the operations to extract data from source electronic health record systems, alignment of the source, and target models on the semantic level and the operations to populate target common data repositories.

**Approach:** In order to separate the activities that are required to transform heterogeneous data sources to a target CDM, we introduce a semantic transformation approach composed of three steps: (1) transformation of source datasets to Resource Description Framework (RDF) format, (2) application of semantic conversion rules to get the data as instances of ontological model of the target CDM, and (3) population of repositories, which comply with the specifications of the CDM, by processing the RDF instances from step 2. The proposed approach has been implemented on real healthcare settings where Observational Medical Outcomes Partnership (OMOP) CDM has been chosen as the common data model and a comprehensive comparative analysis between the native and transformed data has been conducted.

**Results:** Health records of ~1 million patients have been successfully transformed to an OMOP CDM based database from the source database. Descriptive statistics obtained from the source and target databases present analogous and consistent results.

**Discussion and Conclusion:** Our method goes beyond the traditional ETL approaches by being more declarative and rigorous. Declarative because the use of RDF based mapping rules makes each mapping more transparent and understandable to humans while retaining logic-based computability. Rigorous because the mappings would be based on computer readable semantics which are amenable to validation through logic-based inference methods.

## Introduction

It is a well-accepted fact that drugs may still have serious side effects (Nebeker et al., 2004), even after they are marketed. Since the scope and duration of clinical trials are limited, postmarketing drug surveillance has been a necessity in order to capture Adverse Drug Events (ADEs). Pharmacovigilance is the science focusing on the detection, assessment, and prevention of the ADEs and any other drug-related problems (World Health Organization, 2002). Historically, drug safety surveillance research in pharmacovigilance has depended on the mandatory reports produced by randomized trials of the industry and the case reports that are voluntarily submitted to the regulatory authorities. As a promising alternative, there is a growing interest in the secondary use of observational healthcare datasets for postmarketing surveillance.

Electronic Health Records (EHR) available as healthcare datasets cover extended parts of the patient medical history and include more complete information about the risk factors compared to spontaneous case reports. Despite their drawbacks such as potential bias (Moses, 1995; Kunz and Oxman, 1998), this broad range of clinical information could be highly beneficial for surveillance studies to complement and strengthen the existing postmarketing safety studies (Suling and Pigeot, 2012; Coorevits et al., 2013). In order to increase the effectiveness, studies should be extendible and make use of the data from various sources. However, healthcare datasets are generally stored in different heterogeneous information models by organizing the data in different formats and making use of local, diverse vocabularies, and terminology systems. In order to utilize data from heterogeneous systems, either the analysis should be tailored for each data model and the underlying terminologies, or transformation to a common data model (CDM) should be performed (Overhage et al., 2012). Without a CDM, researchers need to develop custom analytical methods that can run on each of these heterogeneous data models, which is costly. In addition to this, it presents significant limitations since detailed knowledge about the disparate data models and underlying vocabularies would be required (Bright and Nelson, 2002). Another drawback of dealing with heterogeneous data sources without a common model is the challenge of reproducibility of results across sites. A CDM not only facilitates the understanding of the analysts, uniform implementation, and reproducibility of results, but also enables large scale systematic analysis over disparate sources by producing comparable results through standard analysis routines (Reisinger et al., 2010). Such CDM based interoperability approach is also studied in the context of data exchange between EHR systems in order to improve interoperability between EHR providers.

The importance of interoperability between EHR systems is obvious. There exists numerous standards such as HL7/ASTM CCD and IHE PCC templates, FHIR Specification^1^, HITSP C32/C83^2^,^3^ components, ISO/CEN 13606 Reference Model^4^ with the aim of providing common representation format for observational EHR data. Such standards foster automated exchange of medical records between EHR systems, and provide a consistent way to represent and update medical records.

FHIR particularly attaches importance to defining set of data formats and resources which comprise the building blocks of majority of interoperability scenarios. This way medical information available in heterogeneous models can be exchanged in structured way on various levels. Inline with its objectives, FHIR is actively developing a framework with the purpose of executing Clinical Query Language^5^ (CQL) over EHR datasets (Jiang et al., 2017). EHR data available in clinical data repositories is manually mapped into common FHIR RDF model, then queries represented in CQL are automatically translated into SPARQL queries to be executed on FHIR RDF graph. However, it is important to note that these models are developed for patient care and are not suitable for research studies (Liao et al., 2015). Initiatives like Observational Medical Outcomes Partnership^6^ develop and publish CDMs in order to enable large scale systematic analysis over observational EHR datasets.

Transformation of observational EHR datasets into CDM narrows the gap between clinical care and research domains as it allows utilization of existing medical summaries for postmarketing safety analysis. Carrying out clinical research studies over existing observational data converted into a CDM has potential advantages over randomized clinical trials such as better generalizability due to broader population, lower cost, and longer time span (Benson and Hartz, 2000); and over case reports suffering from differential reporting, underreporting, and uneven quality problems (Piazza-Hepp and Kennedy, 1995; Brewer and Colditz, 1999; Wysowski and Swartz, 2005; Furberg et al., 2006). In addition, utilization of EHR datasets for postmarketing safety analysis can significantly reduce the time needed to assemble patient cohorts and thus analysis time compared with the years needed to recruit patients and collect case reports (Liao et al., 2015).

It is important to transform the source EHR data into to the CDM easily and accurately as the outcome of the clinical studies heavily depends on the quality of the transformation. ETL is the widespread transformation method where the source and target repositories have different representation formats. An ETL process is composed of three main steps that are dedicated respectively to (i) extract the data from the source repository, (ii) transform the data to the target representation format, and (iii) load the transformed data into the target repository. In order to set up an ETL operation, however, one has to understand the physical data models of both repositories at a detailed level in addition to the domain knowledge required to map the source model to the target model. In accordance with this, it is a well-known fact that the transformation process requires a significant amount of expertise and time considering the complexity of existing information models in both clinical care and research domains (Reisinger et al., 2010; Overhage et al., 2012; Zhou et al., 2013; Matcho et al., 2014). The level expertise required to extract, transform, and manage the EHR data is known to be among the biggest obstacles to utilize EHR datasets in clinical research (Liao et al., 2015).

Existing work on semantic interoperability between EHR systems mostly rely on ETL operations to transform individual EHR datasets into the common representation format. For instance, Santos et al. (2010) proposes a logical EHR model based on ISO/CEN 13606 Reference Model for an integrated EHR service. Proposed interoperability scenario maintains a centralized EHR warehouse utilizing ISO/CEN 13606 and requires individual EHR systems to implement mechanisms for data exchange with the centralized repository. Each EHR provider needs to setup an ETL operation in such a scenario.

Poor documentation, high number of data sources, and ever-evolving nature of data models make designing ETL processes harder; which even made researchers to utilize conceptual modeling frameworks like UML and BPMN in order to address the complexity of the process (Tziovara et al., 2007; El Akkaoui et al., 2013). Furthermore, data quality problems driven by the lack of systematic validation and automated unit testing are important dissuasive factors for using ETL (Singh and Singh, 2010). Oliveira and Belo (2012) points out the lack of a simple and rigorous approach for validation of ETL processes before performing the complete transformation job.

The main goal of this study is to introduce an alternative data transformation methodology addressing the limitations of the traditional ETL approach for the secondary use of EHRs in postmarketing surveillance. The proposed method employs modular components for each step of a data transformation process (i.e., extract, transform, and load). While these steps do not have a clear separation in traditional ETL design tools, we define well-defined boundaries and make use of semantic web technologies for all transformation steps: for the representation and execution of transformation rules as well as representation of the source and target data models. To the best of our knowledge, our proposed methodology is the first automated approach that utilizes semantic web technologies both for description and verification of the target data model as well as the transformation process itself.

As a case study, we validate our methodology by transforming data from two different EHR systems to Observational Medical Outcomes Partnership (OMOP) CDM, one of the most adopted and well-known CDMs for postmarketing safety studies. Throughout this case study, we model OMOP CDM as an ontology, define and execute semantic transformation rules, and employ a software to populate a relational database keeping OMOP CDM data instances and terminology systems. Moreover, we apply descriptive analysis routines from OMOP^7^ to compare the occurrences of specific records in source and target databases to validate the transformation process. Lastly, we apply Temporal Pattern Discovery, a statistical method to recognize patterns in observational data for understanding the post market effects of drugs (Norén et al., 2013).

## Materials and methods

**Figure 1:**
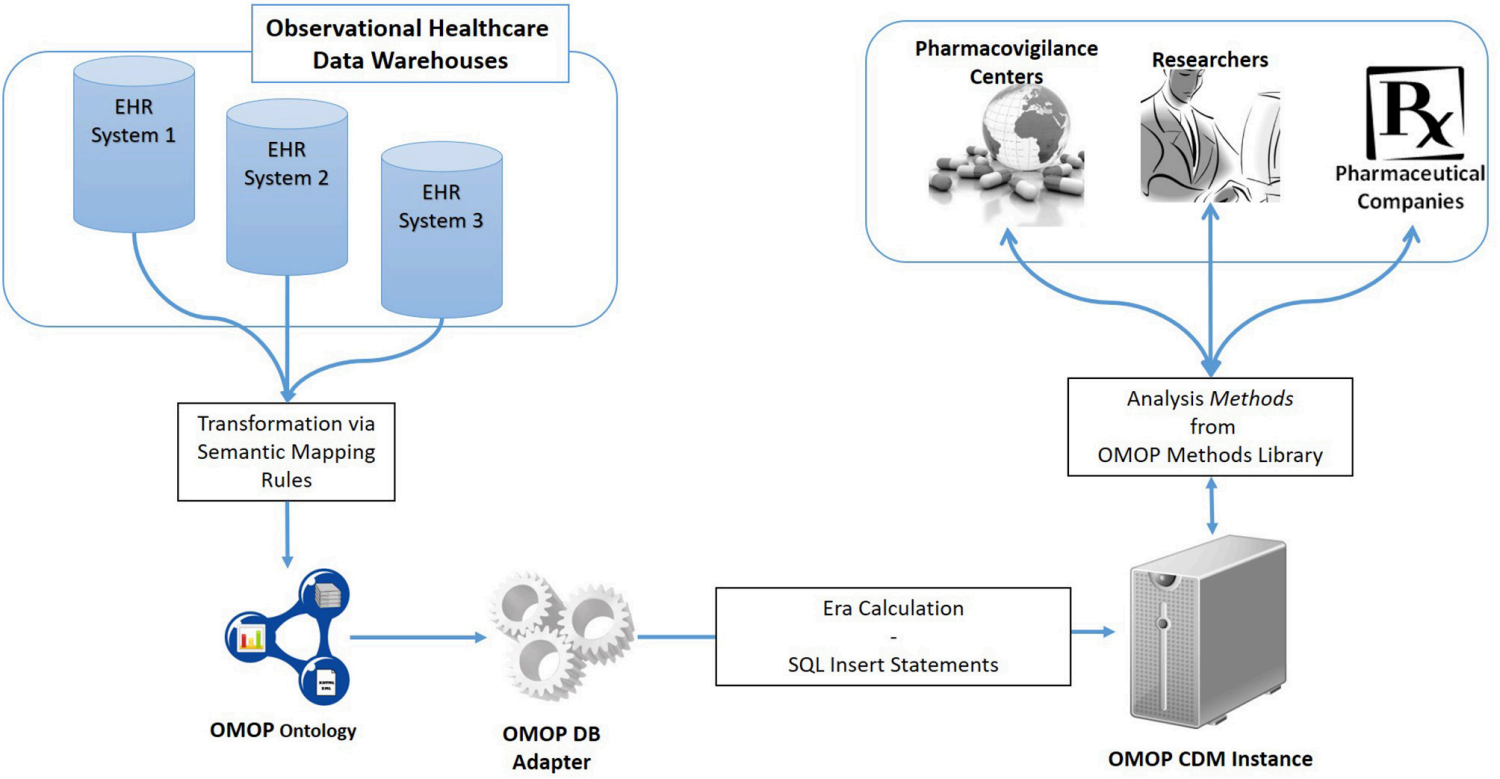
Overview of the semantic transformation methodology—population of a CDM repository from disparate EHR datasets through semantic mapping rules.

An overview of the proposed semantic transformation methodology is presented in Figure 1. The process is handled in three steps in analogous with the traditional ETL approach; but with clearly separated activities in each step. Initially, EHR data is retrieved in RDF format from the underlying EHR systems. This data is provided as input to the semantic transformation rules which are created by domain experts to express semantics of the data transformation. Reasoning engine generates RDF instances expressed in the ontological representation of the target CDM as a result of the reasoning process. This is an intermediate representation processed by a software module to automatically populate the target database. Once the target database is populated, standard analysis methods designed for the target CDM can be seamlessly executed.

### Design and implementation of the transformation methodology

The generic methodology described above is validated by transforming the observational data from two different EHR systems to OMOP CDM. One EHR source, called LISPA, is a regional data warehouse in Italy and the other one, called TUD, is an EHR database of a university hospital in Germany.

#### Retrieval of EHR data in RDF format

We follow different ways for the two EHR sources to get the data in RDF format. TUD case is trivial as the EHR system there provides the data in RDF format already. However, the LISPA system provides medical data represented in HL7/ASTM Continuity of Care Document (CCD)^8^ / IHE Patient Care Coordination (PCC) templates^9^. A tool called Ontmalizer is used to convert data, which is received in native XML representations of CCD/PCC templates, into RDF instances. The resultant RDF is a one-to-one correspondence of the CCD/PCC templates. Either obtained as it is or via a transformation process, the RDF representation at the end of this step reflects the data sources' own native formats. As depicted in Figure 1, heterogenous RDF representations are transformed into a common format via semantic mappings in order to perform the same set of analytic routines on a unified clinical research database.

XML based EHR data representation standards are widely used e.g., HL7/ASTM CCD and IHE PCC templates, FHIR Specification^10^, HITSP C32/C83^11^,^12^ components, ISO/CEN 13606 Reference Model^13^. Although some standards like FHIR are actively working on developing tools to transform existing data in RDF format, XML is still the most dominant representation used by EHR providers. Furthermore, these efforts mostly focus on developing *ad-hoc* mappings for a single model (Jiang et al., 2017). Therefore, having the capability of converting any XML data compliant with an XML Schema Definition (XSD) makes Ontmalizer a convenient tool to convert EHR data represented in these standards into RDF format. Medical summaries transformed into RDF format can be provided as input to the proposed semantic transformation framework. On the other hand, legacy EHR systems are based widely on relational database management systems (RDBMS). There are also efforts for accessing relational databases as virtual, read-only RDF graphs and creating dumps of the databases in RDF format (Hert et al., 2011; Michel et al., 2014). It is also possible to utilize mapping languages from relational databases to RDF model to view the relational data in the RDF format (Bizer and Seaborne, 2004). Utilization of such tools expedites the adaptation of the proposed semantic transformation approach by means of automated RDF generation.

#### Transformation of EHR data in RDF format to OMOP ontology instances

Observational Medical Outcomes Partnership (OMOP) is a public-private partnership funded and managed through the Foundation for the National Institutes of Health (NIH), with the overall aim to improve the safety monitoring of medical products. For this purpose, OMOP conducts comparative evaluation of analytical methods for safety surveillance of longitudinal observational databases across a spectrum of disparate data sources. OMOP methods are based on a model where data from various sources is extracted and transformed to a common structure for further analysis—OMOP CDM. Use of such common data model eliminates the need for tailoring analytical methods for each data source. Methods in OMOP Method Library^14^ can be directly applied once the transformation into OMOP CDM is performed.

**Figure 2:**
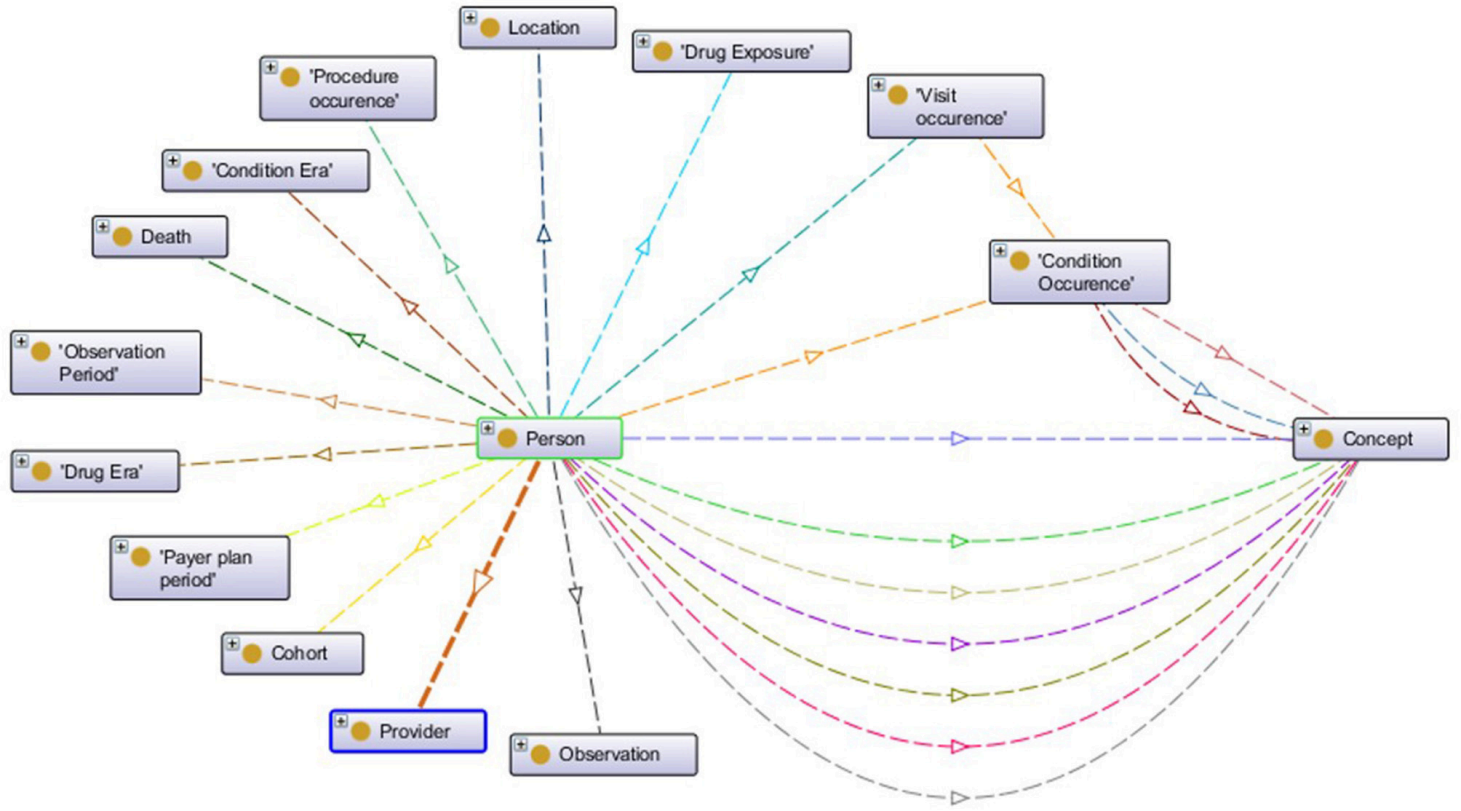
Visualization of OMOP ontology constructs.

Unlike the aforementioned EHR data standards that have an XML Schema defining the structure, OMOP CDM is defined through entity-relationship diagrams and SQL scripts. Since semantic conversion rules are based on RDF technology, we have created the ontological representation of OMOP CDM in Web Ontology Language^15^ (OWL). For each construct in the original OMOP CDM, an OWL construct has been created according to mappings as given in Table 1. Generated OMOP ontology, depicted in Figure 2, is isomorphic to the original OMOP CDM. Similar to the OMOP CDM, OMOP ontology is constructed around the “Person” main class with links to other classes representing various healthcare entities. The complete ontology is available online^16^.

**Table 1:** Mappings of ER constructs to OWL resources.

**ER constructs**	**OWL resources**
Entity	owl:Class
Attribute	owl:DatatypeProperty
Relationship	owl:ObjectProperty

**Figure 3:**
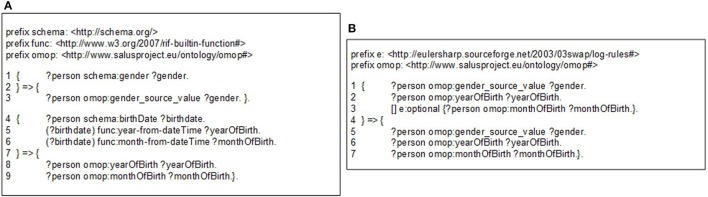
**(A)** A sample semantic conversion rule for person gender and birthdate, **(B)** A sample filtering rule to check existence of gender and year of birth.

The OMOP ontology introduces a level of abstraction and serves as a middle layer in the transformation process of the source EHR data model into the OMOP CDM. Semantic mapping rules are RDF-to-RDF transformation rules such that they take the patient data available in the RDF format and convert them into OMOP ontology instances by hiding the technical details of both the source EHR systems and the OMOP database. Proposed transformation framework hides the technical details of the transformation process and enables user to focus on expressing transformation semantics through abstract mapping rules. Figure 3A shows sample semantic mapping rules that convert semantic EHR data to an OMOP ontology representation. The mapping rules from local domain models to OMOP CDM have been created in co-production among the linked data experts of technical partners, clinical experts of the EHR data sources, and the safety analysts of the pharmacovigilance partners. The rules were finalized after a few iterations and the formalization of the rules were realized by the linked data experts.

The mapping rules are expressed with Notation 3 (N3)^17^ logic, a language developed by semantic web community as an alternative non-XML, human readable serialization for RDF models. These mappings rules can be executed by a N3 reasoner, such as Euler yet another proof Engine (EYE)^18^. A mapping rule in Figure 3A (Line 1–3) depicts how gender expression from the source ontology to is mapped to OMOP ontology. This rule simply generates a one-to-one mapping between gender codes of source and target models. A more complex rule presented in Figure 3A (Line 4–9) demonstrates how birthdate value of patient is broken into components and used to populate corresponding fields in OMOP ontology. The complete set of rules is available online^19^.

Through filtering rules, our semantic rule based approach makes the transformation process easier to validate compared to the traditional ETL approach. Filtering rules can easily be represented in N3 logic and enforced by the N3 reasoner during rule execution. For example, OMOP CDM specifies gender and year of birth as mandatory, while month of birth as optional. Figure 3B depicts the representation of such constraints using *OPTIONAL* construct.

**Figure 4:**
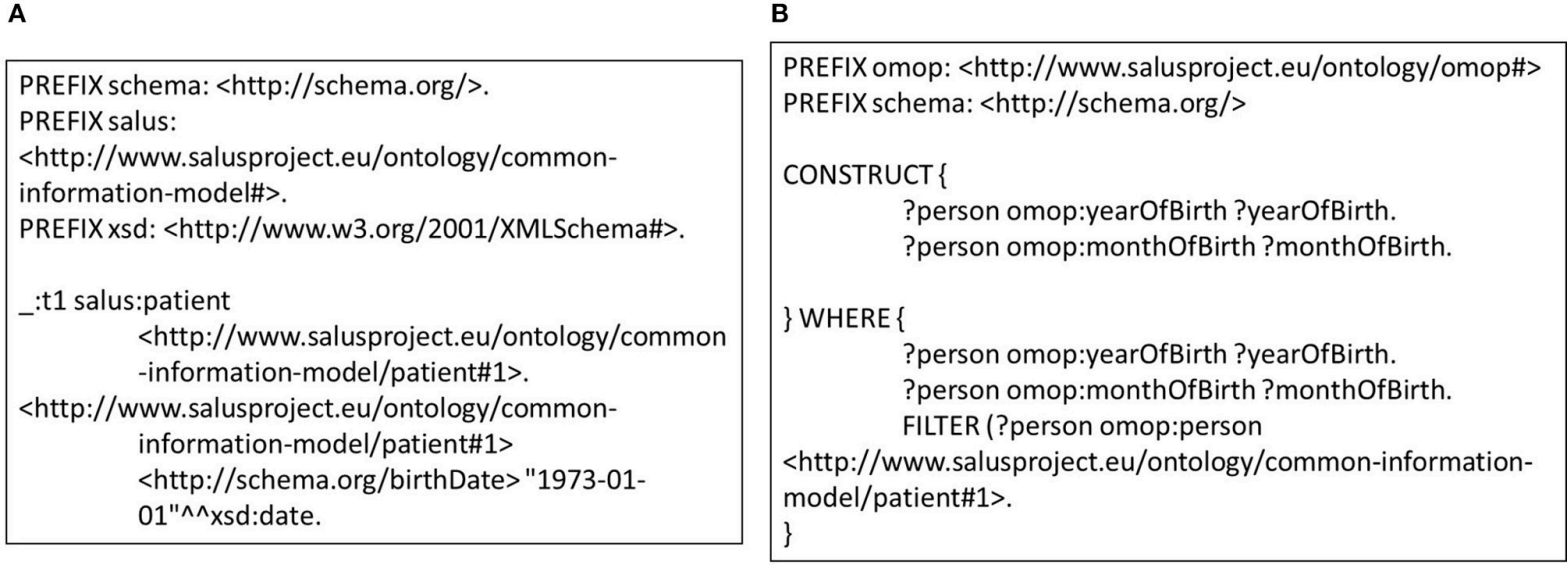
**(A)** Small portion of a patient data that covers the gender and the birthdate. **(B)** A sample unit test for semantic transformation rules.

This approach also enables definition of transformation rules in a modular way for data entities in varying granularities e.g., for concept codes, dates, medications, or observations. Individual rules that are defined at concept code level can be extended and combined in a bottom-up manner in order to define mapping rules for more complex entities such as condition or person. Furthermore, these building blocks can easily be re-used across various versions and models. Such an approach does not only make transformation process easier to maintain compared to the traditional ETL approach, it saves domain experts from the tedious job of developing entire set of transformation rules for every data model. In addition, modularity is a critical enabler for validation of the transformation rules individually as well as the entire process holistically. Exploiting this characteristic, we developed unit tests for each rule. Figure 4A shows a very small portion of a person's EHR data; simply the birthdate of the person. On the other side, Figure 4B shows the unit test checking the validity of the rule in Figure 3A. Running this rule on the test data (Figure 4A) produces the outcome, on which the unit test is applied. The unit test, defined as a *CONSTRUCT* query, aims to re-generate all the expected data elements from the outcome described above. Carrying out the data transformation with the semantic conversion rules and filtering rules by a N3 reasoner does not only make the transformation process more explicit and verifiable as depicted, but also makes it provable. The EYE reasoning engine generates logic based proofs for its reasoning process. A proof records the actions such as the data extractions and inferences that lead to the conclusions of a reasoning process. The proof is checked to build trust on the reasoning process.

#### Populating the OMOP CDM database with OMOP ontology instances

OMOP maintains a library of statistical methods that are developed by researchers and data analysts in pharmacovigilance in order to enable researchers to conduct similar analyses on disparate data sources and obtain comparable results. Standardized methods from the OMOP Methods Library are designed for OMOP CDM, which specifies a relational database schema to define the structure as well as the standardized vocabulary to define the content. Therefore, a relational database instance implementing the OMOP CDM should be populated from the RDF based OMOP ontology instances which are produced during the previous step. At this step, first the condition and medication records along with the persons are loaded to the OMOP database.

One of the major issues at this phase is the mapping of code systems used in the source and target domains. In our case, we have three different code systems for conditions; ICD-9-CM, ICD-10-GM used in our data sources LISPA and TUD, and MedDRA used by clinical analysts for the selected analytical methods. During the transformation, we preserve the original code systems of EHR sources i.e., ICD-9-CM and ICD-10-GM. However, the built-in OMOP vocabularies do not include ICD-9-CM and ICD-10-GM. They were pre-installed into the OMOP CDM before the transformation process. Afterwards, once the analyst initiates an analysis with a MedDRA term, equivalent ICD-9-CM or ICD-10-GM terms are extracted from the Terminology Reasoning Service (Yuksel et al., 2016) and used in the rest of the analysis. By means of terminology reasoning service, our semantic transformation approach is able to run analytical methods which employ a different code system than original data source. For medications, all parties use ATC code system in our pilot scenario. On the other hand, we mapped the gender codes during the transformation time from HL7 Administrative code system to OMOP gender codes using the same Terminology Reasoning Service. Note that use of specific code system is orthogonal to transformation approach presented in this paper and our approach can be integrated with any code system through a Terminology Reasoning Service. Our Terminology Reasoning Service employs original terminology systems as formalized ontologies whose hierarchical relationships are represented with “skos:broader” property, and several reliable resources are utilized for the mappings across different code systems, again as semantic relationships. For example, OMOP project provides mapping of a selected subset of ICD-9-CM and ICD-10-CM codes to SNOMED-CT Clinical Findings; these are represented via the “salus:omopMapping” property. IMI PROTECT project created on ontology called OntoADR, which represented mappings between MedDRA and SNOMED-CT codes; these are represented via the “salus:protectCloseMatch” property in our Terminology Reasoning Service. By using such reliable relationships, we apply a series of terminology reasoning rules, again implemented on top of EYE, to deduce all the code mappings that we need in our use case in advance. These inferred code mappings are fed to the Terminology Reasoning Service, so that the OMOP queries with MedDRA codes are expanded with corresponding codes from ICD-9-CM and ICD-10-GM while being executed on top of source EHR data. Further details about our terminology reasoning approach can be found in Yuksel et al. (2016).

The OMOP DB Adapter, the software module dealing with the database-specific details, consumes the OMOP ontology instances and populates the OMOP CDM instance. In addition to the population of the OMOP CDM instance, it is also responsible for the calculation of condition and drug eras which are the chronological periods of occurrence of conditions and exposure of the drugs, respectively. OMOP CDM is designed to combine independent exposures of the same drug or occurrences of the same condition into a single era through a persistence window. Persistence windows represent the allowable timespan between the exposure of the same drug and occurrence of the same condition. As indicated in the OMOP CDM specification (OMOP CDM, 2013), the length of a persistence window is set as 30 days. In case of medications, if there is less than 30 days between consecutive exposures of the same drug, then a drug era is created and both exposures are merged. If there is no other drug exposure in the persistence window, then an era is created from that single exposure. All drug exposures are either merged into an already existing drug era or constitute a drug era itself so that all exposures are associated with drug eras. Similarly, all condition eras are calculated using the condition occurrence records. The era calculation is performed on the fly during the generation of SQL Insert statements from the OMOP Ontology instances. The OMOP DB Adapter produces one-to-one correspondence of the OMOP Ontology instances that are produced in the previous step and populates the target OMOP CDM instance in fully automatized manner.

### Evaluation design

The methodology that we introduce in this study has been built within the SALUS interoperability framework. SALUS (Laleci Erturkmen et al., 2012) is a research project that aims to create a semantic interoperability layer by aligning the source and target data models as well as the code systems used to represent healthcare concepts (e.g., drugs and conditions) in order to enable the secondary use of EHR data for clinical research activities. SALUS semantic interoperability layer accepts eligibility queries and returns resultant patient summaries as instances of a RDF based data model, i.e., the Common Information Model (CIM) of SALUS. The process of getting EHR data in RDF format is realized through SALUS interoperability framework (section Retrieval of EHR Data in RDF Format). The OMOP ontology is derived from these SALUS CIM conformant patient summaries by means of semantic mapping rules (section Transformation of EHR Data in RDF Format to OMOP Ontology Instances) and loaded into the target OMOP CDM instance by means of the OMOP DB Adapter (section Populating the OMOP CDM Database With OMOP Ontology Instances). Through real-world deployments of the SALUS in TUD and LISPA, we aim to show that proposed semantic transformation framework can effectively populate an OMOP CDM instance and expedites clinical research activities on heterogeneous data sources.

We perform two main activities for the evaluation of this methodology. The first is to compare descriptive statistics from the source and target databases in order to verify the correctness of the transformation process, which is a common method in literature to assess to quality of the data transformation process (Santos et al., 2010; Overhage et al., 2012; Zhou et al., 2013; Jiang et al., 2017). The OMOP team created the Observational Source Characteristics Analysis Report^20^ (OSCAR) to characterize the transformed data represented in the OMOP CDM format. OSCAR provides a structured output of the descriptive statistics for the data represented in the OMOP Common Data Model. Such systematic data extraction facilitates comparison of source and target databases for quality assessment and plays a key role in validation of transformation into the OMOP Common Data Model. Since OSCAR is not available in pilot settings of SALUS (i.e., TUD & LISPA), we developed set of *ad-hoc* SQL queries to calculate statistics similar to OSCAR. Those statistics were extracted for two specific patient cohorts in addition to the entire dataset, namely “Patients with acute myocardial infarction (AMI) occurrence” and “Patients with nifedipine exposure.” The main objective of this activity is to assess the quality of the transformation process and show that the resultant database preserves the characteristics of the source database. Similar to original OSCAR, extracted statistics do not contain any personal level data and can be shared.

The second activity is to show that clinical researchers are able to detect and evaluate potential signals from the resultant OMOP CDM instance which is populated using the proposed semantic transformation methodology. One such scenario starts with the analysis of the whole OMOP CDM instance for potential, causally related drug—adverse drug event pairs. In this step, the analysts may choose to investigate one or more (possibly all) drugs against one or more (possibly all) ADEs. Main objective is to show that existing analysis methods from literature can directly be applied on resultant database, eliminating the need for tailoring data analysis for each source data model.

**Figure 5:**
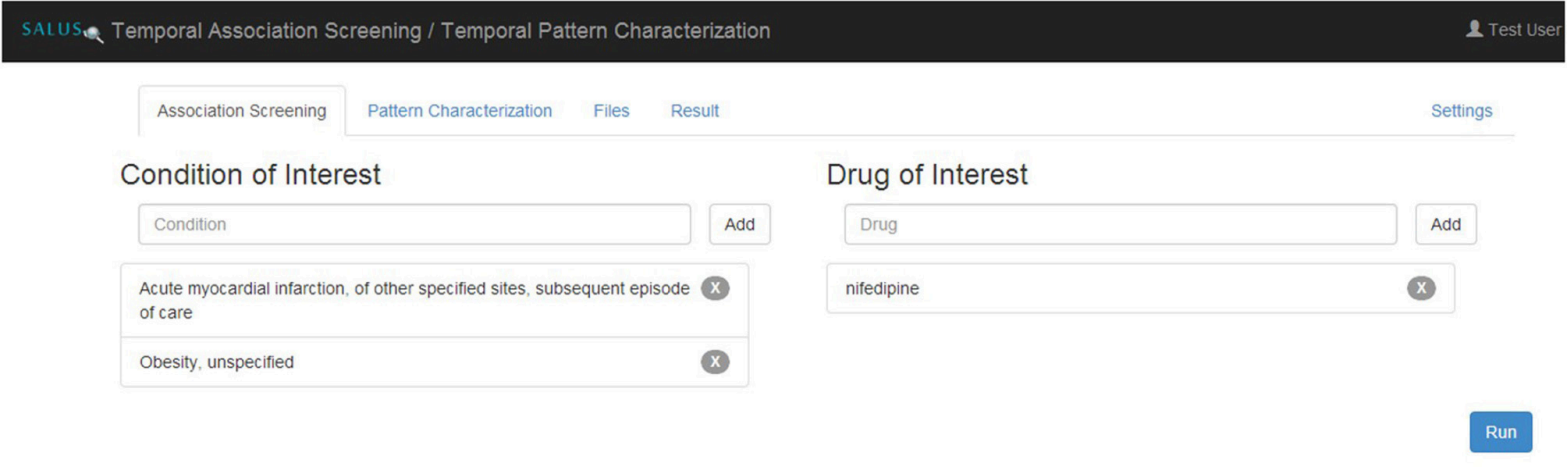
Specifying eligibility criteria in TAS.

Temporal Association Screening (TAS) tool of the SALUS project realizes this scenario by running IC Temporal Pattern Discovery proposed by Noren (Norén et al., 2013) from the OMOP Method Library. First, the analyst specifies drugs and conditions of interest in ATC and MedDRA terminologies, respectively as depicted in Figure 5. TAS tool queries the Terminology Reasoning Service in order to retrieve corresponding codes in the target data model. After selecting the ICD-9-CM or ICD-10-GM correspondences of the conditions coded by the analyst, Temporal Pattern Discovery is carried out with the ICD codes on OMOP CDM. In order to assess the feasibility and usability of the populated OMOP CDM Instances, the TAS tool is used to investigate drug-condition pairs, i.e., atorvastatin— rhabdomyolysis or nifedipine—acute myocardial infarction (AMI). In addition to the descriptive statistics calculated on the entire dataset, we also report the results related to patient cohort with nifedipine or AMI.

**Figure 6:**
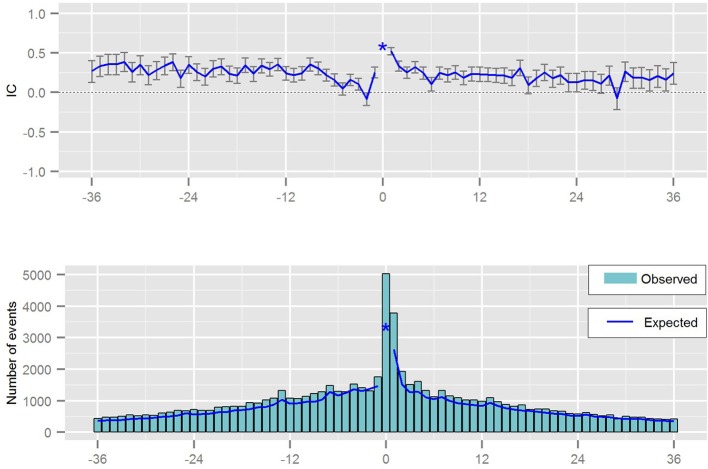
Chronograph visualizing the temporal pattern between a prescription of a drug and the occurrence of a medical event.

The statistical measure derived from the Temporal Association Screening tool is a measure of disproportionality taking some confounders into account. The result of the analysis is presented graphically, via chronographs, to further analyze a specific drug-event pair visually as presented in Figure 6. Analyzing a chronograph gives the safety analyst a visual representation of the empirical basis for a possible association between a drug prescription and an event (Norén et al., 2013).

**Figure 7:**
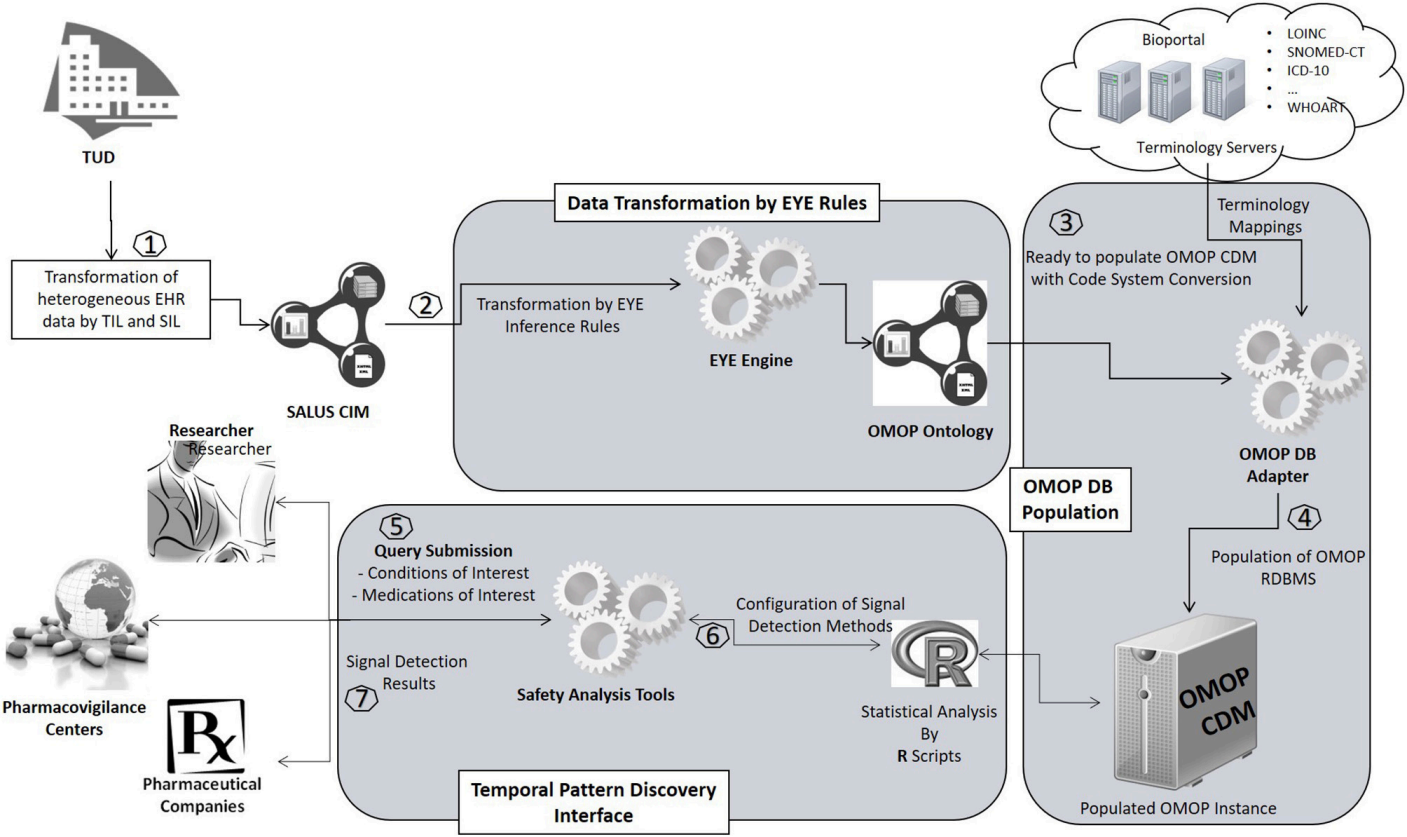
Implementation of the proposed framework in real-world settings.

Our semantic transformation framework has been deployed on two different EHR systems of the pilot sites of the SALUS project, namely LISPA and TUD. LISPA database includes anonymized data of ~16 million patients with over 10 years' longitudinal data on the average whereas TUD EHR System contains ~1 million patient records. Figure 7 depicts the deployment of the proposed framework on the two SALUS pilot sites.

## Results

In this paper, we present the evaluation results obtained from the TUD data warehouse for showing the correctness and accuracy of the proposed transformation methodology.

The number of total patients in the original TUD data warehouse is 893,870 and 95.66% of those patients were accurately translated to OMOP CDM instance. The number of patients in nifedipine cohort accounts for 0.013% of the total TUD patients whereas the number of patients in AMI cohort corresponds to 0.287% of the population. All 113 patients in original nifedipine cohort were successfully preserved during the transformation process and corresponding patient entities were created in the target database. However, six out of the 2,562 patients in the original AMI cohort could not be transformed into the OMOP CDM instance. Table 2 provides the statistics on the number of patients obtained from both source and target databases.

**Table 2:** Patient counts in original and transformed databases.

	**TUD data warehouse**	**Populated OMOP CDM instance**	**Percentage**
Number of patients	8,93,870	8,55,101	95.66
Number of patients in nifedipine cohort	113	113	100
Number of patients in AMI cohort	2,562	2,556	99.77

Detailed investigation on the patients who could not be transferred to the OMOP CDM instance reveals that those patients were left out by the OMOP DB Adapter intentionally for two reasons. First, the patients without a birthdate were not imported since the year of birth is a mandatory field in OMOP CDM (see Figure 4B), which shows the effectiveness of our filtering rules in preserving data integrity. Second, the TUD data warehouse contains several medical cases without any start date; patients associated with those could not be transferred either. All 855101 patients with valid a birthdate and valid medical conditions were successfully transformed into OMOP CDM and loaded into the target database.

**Figure 8:**
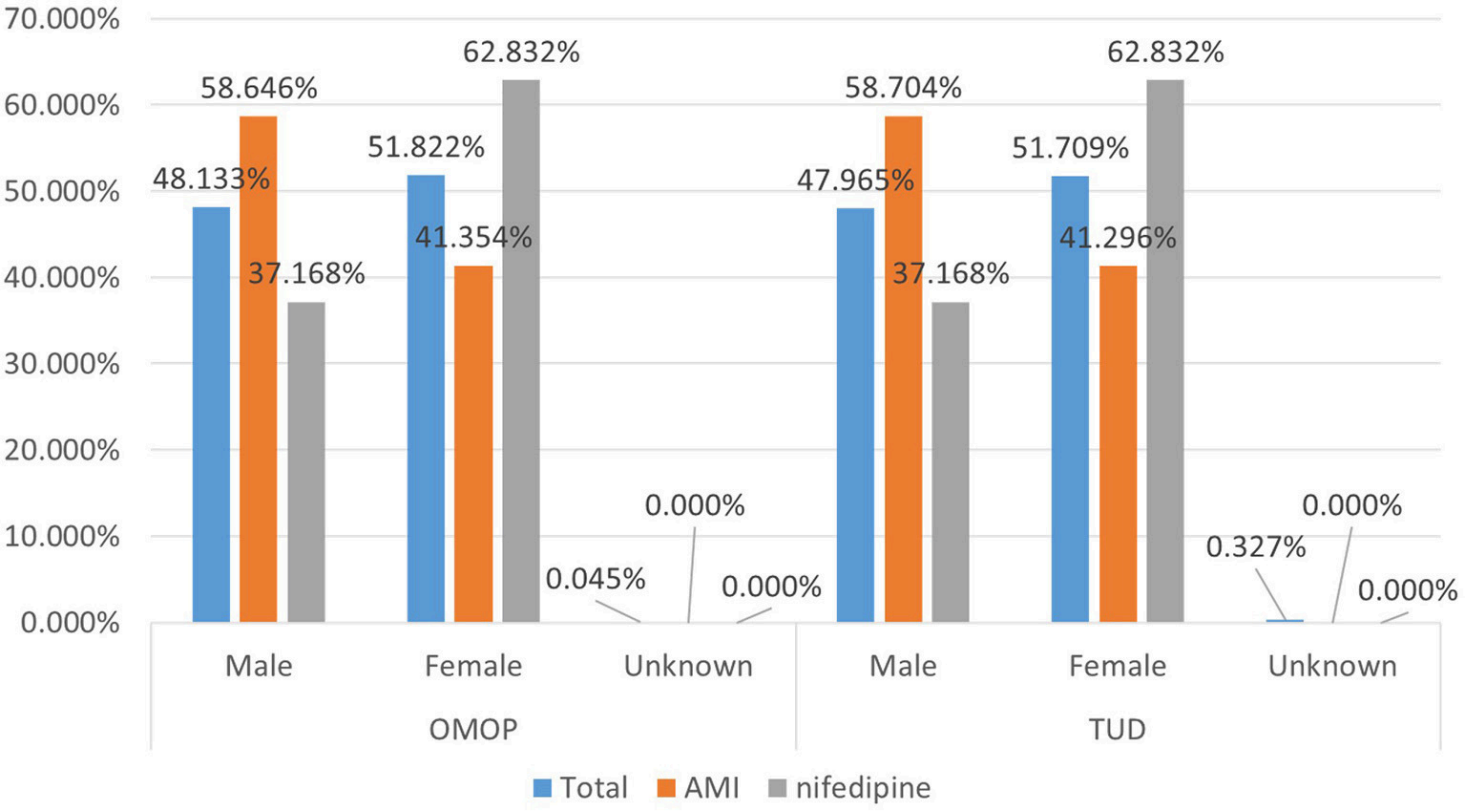
Demographic summary of AMI and nifedipine cohorts in original TUD and populated OMOP database.

Demographic characteristics of the patient population were preserved to a great extent during the transformation. Average age statistics of both gender groups and the whole population are presented in Table 3. Figure 8 presents the gender distribution of the selected cohorts in both original TUD (source) and populated OMOP databases (target). Table 4 compares the nifedipine exposures and AMI occurrences in those databases. In line with the previous findings, both results point out the substantial similarity between two databases; hence the quality of data transformation.

**Table 3:** Patients' age statistic in original and transformed databases.

	**TUD data warehouse**	**Populated OMOP CDM instance**
Average age (overall)	50.723	50.877
Average age (male)	49.917	49.879
Average age (female)	51.791	51.848

**Table 4:** Patient counts of selected cohorts in original and transformed databases.

	**TUD data warehouse**	**Populated OMOP CDM instance**
**NIFEDIPINE**		
Total exposures	494	494
Average exposure per patient	4.371	4.371
**ACUTE MYOCARDIAL INFARCTION**		
Total occurrences	6708	6705
Average occurrence per person	2.618	2.623

**Figure 9:**
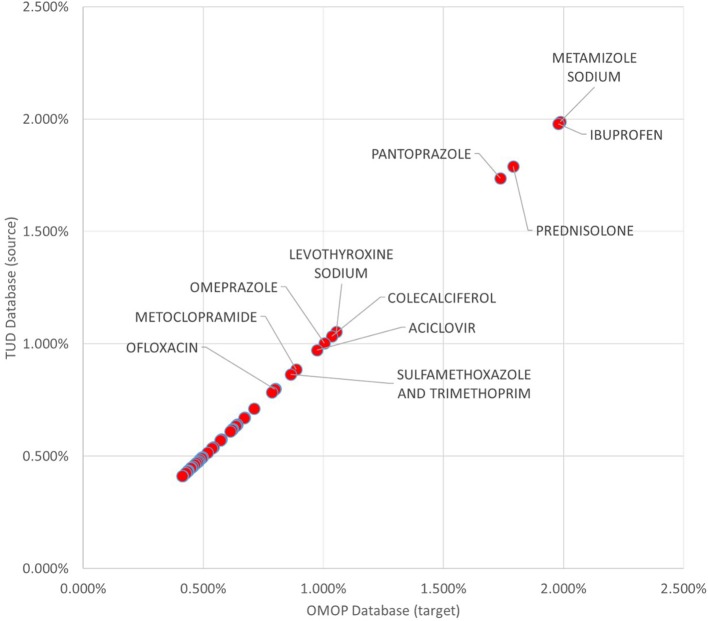
Proportion of Top 50 medications to whole exposures in TUD and transformed OMOP databases.

99.93% of all medication records in the drug exposure table were transformed to the OMOP CDM instance. Top 100 most frequent medications accounts for 52.55% of the drug exposure data and 96.87% of those were correctly reflected in the populated OMOP CDM instance. Investigation on the unmapped drug exposures points out the previously discussed filtering conditions. In Figure 9, 50 most frequent medications with their incidence rates in source TUD database and target OMOP CDM instance is presented. It shows that incidence rates of medication in both databases are well-aligned.

**Figure 10:**
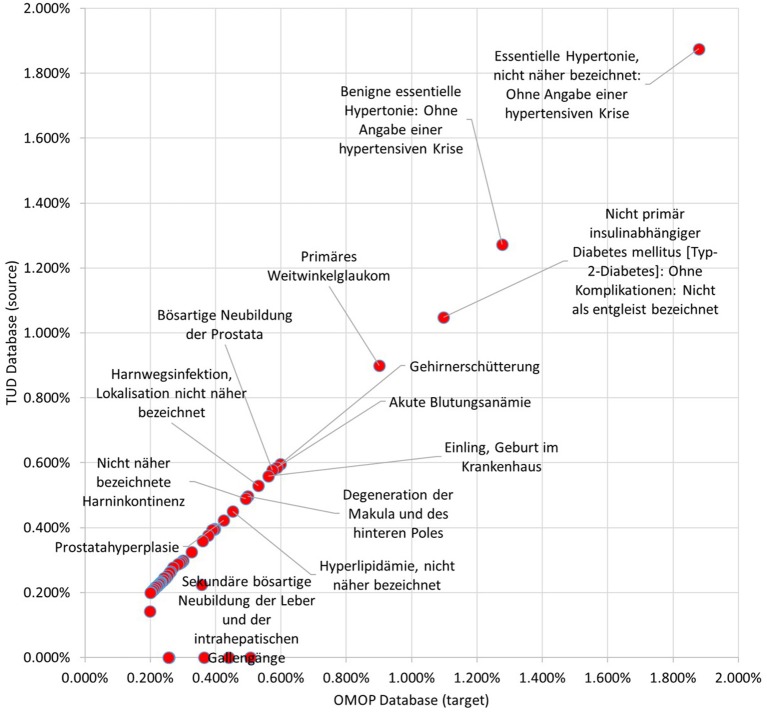
Proportion of Top 50 conditions to whole occurrences in TUD and transformed OMOP databases.

99.59% of the condition occurrences were successfully transformed. Top 100 most occurring conditions made up 29.96% of all conditions data and 94.55% of these occurrences were mapped to OMOP CDM instance successfully. Incidence rates of the 50 most occurring conditions in both databases are presented in Figure 10. It also presents a linear trend line as medication incidence rates except a few conditions. The cause of these abnormalities is the differences between the vocabularies of the source and target databases. In TUD, the German modification of ICD10, which is not part of the standardized OMOP Vocabulary, is used to annotate conditions. Therefore, any conflict between these code-lists were handled by the OMOP DB Adapter during the transformation. For instance, the number of conditions which are not in the top 100 in original data were mapped to “Essentielle (primäre) Hypertonie”—“I.10-” and created inconsistency between the original and transformed data. However, this problem effects a statistically negligible portion of the condition records as described previously.

## Discussion

This research proposes a semantic conversion approach over the prevalent ETL approach to use observational health records in postmarketing safety studies, and the convenience of the proposed approach for clinical researchers is shown through a real-world deployment. The proposed semantic transformation methodology enables data sources to be transformed into a target common data model by only providing abstract mapping rules and eliminates the effort spent to address database specific details. We implement the proposed methodology based on the OMOP common data model due to rich set of standardized analysis methods and its prevalence in pharmacovigilance research. Unlike the traditional ETL approach, the use of abstract mapping rules enables researchers to focus on the semantics of the data models rather than designing the entire transformation pipeline. Furthermore, abstraction of the semantic and technical steps fosters modularity and re-use, making the proposed framework a scalable, verifiable, and provable approach.

Semantically mediating all the patient data, terminology systems, and the mappings among terminology systems in formalized representations in a knowledge base allows us to improve the quality of data and extend the capabilities of our end-user tools via introduction of new rules easily. The patient data in the EHR systems is not perfect most of the time; it can be incomplete or erroneous. With our approach, for example we are able to insert a new rule to infer diabetes diagnosis by checking the existence of specific medications (e.g., metformin) and laboratory test results (e.g., high glycosylated hemoglobin [HbA1c] result) when diabetes is not explicitly recorded in the list of diagnoses of a patient due to incomplete records. Another advantage of our semantic transformation approach is that, terminology code mapping is decoupled from the content model transformation and all the original codes from the local data (i.e., the source of truth) are preserved in the transformation process so that no meaning is lost in translation. The code mapping is handled dynamically at run time by query expansion enabled by our Terminology Reasoning Service, which is continuously and independently being enriched with validated / invalidated code mappings. In standard ETL approach though, code mapping is hardwired in the ETL scripts and hence the source of truth is lost in the target data. Even when a single new code mapping is introduced by the terminology experts, the ETL scripts have to be updated and re-run on the whole source data.

Use of semantic conversion and filtering rules based on N3 logic enables reasoning engine to generate proof records, which in turn can be automatically checked and verified by the same engine in order to build trust on the transformation process. Thus, correctness of the entire transformation process can be verified in bottom-up manner by combining the proof records generated for each conversion and filtering rule. In addition, storing mapping rules in a machine-processable format combined with robust versioning makes maintenance easier in case of changes in data sources and facilitates adaptation of new models by increasing re-use of existing rules.

On one hand the methodology, specifically the transformation process, is easier to understand as the mapping rules are well-documented and transparent to the user; easier to maintain (thus easier to generalize and re-use) as the rules are linked to the relevant data entities and stored in a machine-processable format; on the other hand, it is more rigorous thanks to fine-grained unit testing and automated verification mechanisms on the filtering and conversion rules.

Recently, standard developing organizations investigate how semantic technologies can be adopted in order to create a formal, machine-processable representation for existing clinical data models. Yosemite Manifesto (2016) positions RDF as “the best available candidate for a universal healthcare exchange language” and asserts that healthcare information should either already be represented in RDF or have mappings to it. HL7 and CDISC groups are in an effort to publish their existing models in RDF. It is an important move toward enabling semantic interoperability in clinical research, inline with our objectives.

FHIR adapted RDF as the third instance representation format in addition to JSON and XML. In addition, FHIR developed Shape Expressions (ShEx) language to formally define data constraints in FHIR RDF Graphs, which serves as a formal language to describe and define instance graphs (Solbrig et al., 2017). Our proposed approach can complement the FHIR RDF and ShEx and makes it possible to extend this RDF based interoperability framework into clinical research domain. One can define abstract mapping rules from FHIR RDF into OMOP Ontology so that observational data represented in FHIR RDF graphs can be directly used by OMOP methods. Combined with ShEx, our N3 logic based reasoner can verify the correctness and the integrity of both source data and the transformation process itself.

With the increasing adoption of these RDF based standards, the proposed semantic transformation framework will possibly be applied on more domains and substitute the onerous ETL based approaches. From a practical point of view, tools like Ontmalizer are convenient to utilize widely used, XML-based EHR standards such as HL7/ASTM CCD and IHE PCC templates into RDF format. Also, technologies to access relational databases as virtual, read-only RDF graphs are another modality to utilize EHR in RDF format (Michel et al., 2014).

Although it is orthogonal to the transformation approach proposed in this study, terminology mapping is an important task which has to be considered during the transformation of EHRs to a particular CDM. Major sources like BioPortal (Salvadores et al., 2013) already publish some mappings between different terminology systems backed by Linked Data principles. Similarly, logical EHR model proposed by Santos et al. proposes a terminology service based on semantic web technologies where existing terminologies are encoded in RDF and OWL languages (Santos et al., 2010). In the proposed semantic transformation approach, these mappings can be exploited directly through a Terminology Reasoning Service and several manual processing tasks including retrieval and preparation of the mappings are ruled out; enabling a seamless transformation process.

## Conclusions

We have developed a semantic transformation framework in order to transform available EHR data represented in proprietary or standard content models into an OMOP CDM database instance to be utilized in drug surveillance studies. The proposed framework addresses the limitations of traditional ETL based approaches and introduces an easy-to-use, modular, and verifiable approach through clear separation of technical and semantic steps of the transformation pipeline. The framework consists of a set of *semantic conversion rules* expressing the semantics of the transformation and transforming the EHR data to the OMOP ontology in bottom-up manner; *the OMOP ontology* as the intermediate semantic representation into which EHR data is transformed; *the OMOP Database Adapter* as the software component realizing the transformation in syntactic level and populating the relational OMOP database based on the OMOP ontology instances. In order to show that both source data and the target instance have identical descriptive characteristics, we executed a systematic descriptive analysis between the source EHR database and the target OMOP database. As a result, we observed that source data is accurately transformed into the target model and the target database instance exhibits the characteristics of the original patient population. More importantly, we executed the Temporal Pattern Discovery Methods from the OMOP Methods Library on the database populated through the proposed framework. In addition to the validation of our methodology on a EHR database, applying on two very different EHR sources indicate a promising result that EHR data residing in different systems conforming to different data models can be unified in the same database through the same methodology and by using common components such as the semantic conversion rules and the OMOP Database Adapter.

## Author contributions

AS and GL: initiated the study design; AP, SG, GL, MY, and AS: realized the implementation of the proposed framework; AP: planned the comparative analyses and conducted the analyses with the help of SG, GL, and AS; authors wrote the first draft of the manuscript together; AP: rewrote new drafts based on input from co-authors. All authors contributed to refinement of the manuscript and approved the final manuscript.

### Conflict of interest statement

All of the authors (AP, SG, AS, MY, and GL) were employed by company SRDC Software Research & Development and Consultancy Corp.

## Footnotes

^1^https://www.hl7.org/fhir/overview.html

^2^https://ushik.ahrq.gov/portals/hitsp/documents/c32?system=hitsp

^3^https://ushik.ahrq.gov/portals/hitsp/documents/c83?system=hitsp

^4^https://www.iso.org/standard/40784.html

^5^http://www.hl7.org/implement/standards/product_brief.cfm?product_id=400

^6^https://www.ohdsi.org/data-standardization/the-common-data-model/

^7^http://semanticommunity.info/Data_Science/Observational_Medical_Outcomes_Partnership#Methods_Library

^8^http://www.hl7.org/documentcenter/public_temp_04729A34-1C23-BA17-0CF62AA9C8C50B56/pressreleases/20070212.pdf

^9^http://www.ihe.net/Technical_Framework/upload/IHE_PCC_Suppl_CDA_Content_Modules.pdf

^10^https://www.hl7.org/fhir/overview.html

^11^https://ushik.ahrq.gov/portals/hitsp/documents/c32?system=hitsp

^12^https://ushik.ahrq.gov/portals/hitsp/documents/c83?system=hitsp

^13^https://www.iso.org/standard/40784.html

^14^http://semanticommunity.info/Data_Science/Observational_Medical_Outcomes_Partnership#Methods_Library

^15^http://www.w3.org/TR/owl-features/

^16^https://cs.uwaterloo.ca/~apacaci/files/omop-cdm-ontology.n3

^17^http://www.w3.org/TeamSubmission/n3

^18^http://eulersharp.sourceforge.net/

^19^https://cs.uwaterloo.ca/~apacaci/files/commonToOmopMappings.n3

^20^http://semanticommunity.info/Data_Science/Observational_Medical_Outcomes_Partnership#OSCAR
